# Influence of a nanoscale coating on plucking fingers and stainless steel on attachment and detachment of *Salmonella* Enteritidis, *Escherichia coli* and *Campylobacter jejuni*

**DOI:** 10.1007/s12223-024-01162-3

**Published:** 2024-04-09

**Authors:** Victoria Blaeske, Felicitas Maria Schumann-Muck, Ahmad Hamedy, Peggy G. Braun, Martin Koethe

**Affiliations:** Institute of Food Hygiene, Leipzig University, An den Tierkliniken 1, 04103 Leipzig, Germany

**Keywords:** Cross-contamination, Food contact surfaces, Nanomaterials, Poultry, Slaughtering

## Abstract

Gastroenteritis caused by *Campylobacter* represents the most common reported foodborne bacterial illness worldwide, followed by salmonellosis. Both diseases are often caused by the consumption of contaminated, insufficiently heated poultry meat. This can result from contamination of the meat during the slaughtering processes. Food contact surfaces like stainless steel or plucking fingers contribute significantly to cross-contamination of poultry carcasses. Modification of these surfaces could lead to a reduction of the bacterial burden, as already proven by successful application in various food industry sectors, such as packaging.In this study, nanoscale silica-coated and uncoated stainless-steel surfaces and plucking fingers were compared on a pilot scale regarding attachment and detachment of *Campylobacter jejuni*, *Salmonella* Enteritidis and *Escherichia coli*.The bacteria did not adhere less to the coated plucking fingers or stainless-steel sections than to the uncoated ones. The coating also did not lead to a significant difference in detachment of *Campylobacter jejuni*, *Salmonella* Enteritidis and *Escherichia coli* from the investigated surfaces compared to the uncoated ones.Our study did not reveal any differences between the coated and uncoated surfaces with regard to the investigated bacteria. In order to achieve a better adaptation of the coating to slaughterhouse conditions, future studies should focus on its further development based on the investigation of specific coating parameters.

## Introduction

Foodborne diseases are a global public health issue for human life and health. According to World Health Organization (2021), 600 million cases of illness are caused each year worldwide by eating contaminated food. In this context, diarrheal diseases are the most prevalent consequences. *Campylobacter* are considered the most frequent bacterial cause of gastroenteritis in humans worldwide, accounting for approximately 95 million cases per year, with *Campylobacter jejuni* (*C. jejuni*) being the most widespread human pathogenic species (Centers for Disease Control and Prevention 2023; World Health Organization 2015). Salmonellosis represents the second most common bacterial intestinal disease, whereby *Salmonella enterica* serovar Enteritidis (*S*. E.) is one of the two most relevant serotypes for humans (World Health Organization 2020). One of the major vehicles of *Campylobacter* and *Salmonella* infections in humans is contaminated poultry meat, which is consumed insufficiently heated (Robert Koch-Institut 2020). Efforts were made to prevent the occurrence of the bacteria, especially in primary production, through European-wide control programmes. Although *Salmonella* illnesses have declined, there are still high numbers of infections, and in the case of *Campylobacter*, the disease rate has stagnated at a constantly high level for years (European Food Safety Authority 2021 and European Centre for Disease Prevention and Control 2022; Statista 2022).

Slaughtering as the subsequent downstream part of the meat-producing process is a further focus for reducing bacterial contamination of poultry meat. Cross-contamination during slaughtering poses a particular risk, as potentially pathogenic material can be transferred from one carcass to the next via contact surfaces such as plucking fingers, guide elements or conveyor belts (Veluz et al. 2012). The most critical points in this context are plucking and evisceration, as these highly mechanised processes can lead to an increased exchange of organic material such as feathers, excreta and intestinal content between machines and carcasses (Gruntar et al. 2015; Rasschaert et al. 2007). Either by the pressure of the plucking fingers on carcasses or by the mechanical action of the evisceration equipment, faeces can leak out and be spilled over equipment surfaces to contaminate subsequent carcasses (Pacholewicz et al. 2015; Zeng et al. 2021). Animal carcasses’ contact surfaces of the slaughterhouse are, therefore, of particular importance with regard to possible cross-contamination and transmission of pathogens (Arnold and Silvers 2000). It is well known that pathogenic bacteria such as *Salmonella* and *Campylobacter* are able to colonise surfaces commonly used in food industry, such as stainless steel, rubber or glass (Chia et al. 2009). Modifying these surfaces to prevent the adhesion of bacteria and facilitate their detachment could contribute to an overall reduction of the bacterial load in the slaughter process. Certain natural surfaces are able to reduce bacterial attachment, mostly due to an ultra-thin nanoscale topography, which is why the development of such a surface is also a promising approach in industry (Ivanova et al. 2012). Initial applications on exposed surfaces based on nanotechnology are already well-established practice both in the medical sector and on packaging in the food industry (Chouirfa et al. 2019; Gallocchio et al. 2016). The most promising way to achieve nanostructuring of slaughterhouse surfaces appears to be the application of a coating, as this can be very specifically adapted to the desired effects and respective conditions with regard to the material composition and the modification of the surface structure. A variety of materials, including synthetic materials such as polyurethane (Bakker et al. 2004) and metals like gold (Díaz et al. 2007), have already been tested for their suitability as coating materials. Another material that is being considered for use in this area is silicon dioxide. In addition to already known advantages such as good biocompatibility and low manufacturing costs, a certain effectiveness against bacteria has also been demonstrated in various studies (El-Shetehy et al. 2021; Rao et al. 2005).

In previous studies, small, tending and partly significant effects of nanoscale silicon dioxide coating were observed for *S.* E., *E. coli*, *Listeria monocytogenes* and *C. jejuni* in a laboratory scale (Blaeske et al. 2023; Hillig et al. 2024; Schumann-Muck et al. 2023a, b). We hypothesised that these effects will also be present in a more practical approach in a pilot plant scale. The aim of this study was, therefore, to investigate the effects of this nanoscale coating on stainless-steel slaughterhouse equipment and plucking fingers on the attachment and detachment of *S.* E., *C. jejuni* and *E. coli* on a pilot plant in a more practical environment. *E. coli* was included in the investigations as an indicator bacterium, as its presence on slaughterhouse surfaces can indicate possible faecal contamination and thus also with *Salmonella* or *Campylobacter* (Foley et al. 2008).

## Materials and methods

### Bacterial material

*S. *E. isolate 19-SA00115, *E. coli* isolate 20-AB00467 and *C. jejuni* isolate BFR-CA-19285 were obtained from the German Federal Institute for Risk Assessment (BfR, Berlin, Germany) and originated from chicken meat samples. They were cryopreserved at the Institute of Food Hygiene Leipzig at -80 °C (Cryobank, Mast Group Ltd., Germany). Fresh working cultures of *S*. E. and *E. coli* were obtained as described elsewhere (Schumann-Muck et al. 2023b) and used for a fortnight. Overnight cultures were grown to a cell density of about 10^8^ cfu per mL as described previously (Schumann-Muck et al. 2023b) except for the addition of bovine serum albumin. *C. jejuni* working cultures were obtained by spreading cryopreserved beads on blood agar plates (Columbia Agar with Sheep Blood Plus; Oxoid GmbH, Germany) for each experimental approach and incubating them microaerobically (85% N_2_, 10% CO_2_, 5% O_2_) for 36 h at 42 °C. Therefore, the TRILAB system (TRILAB; Jenny Science AG, Switzerland) was used. The bacterial colonies from these plates were then stirred into Brain Heart Infusion Broth (BHI Broth; TN1216; sifin diagnostics GmbH, Germany) and incubated without agitation microaerobically for 16 h at 42 °C to a cell density of about 10^8^ cfu per mL.

On-site at the pilot scale slaughtering unit, the suspensions of *S*. E., *E. coli* and *C.* *jejuni* were combined in a 1:1:1 ratio for every experimental approach to obtain a pool of target organisms.

### Surfaces

The experiments were carried out in the technical centre of the German Federal Institute for Risk Assessment (BfR, Berlin, Germany). Commercial plucking fingers made of thermoplastic rubber with the following data were used: 20 mm bore diameter; hard version; total length, 97.5 mm; (Westfalia Werkzeug company GmbH & Co. KG, Germany). Before being used in the experiments, the plucking fingers were pretreated by cleaning and sterilisation. For this purpose, they were first placed in 5% Decon solution (Decon™ Decon90, Fisher Scientific GmbH, Germany), then rinsed with distilled water and finally degreased in a 95% 2-propanol solution (Carl Roth GmbH, Germany). After a further rinse with distilled water, they were first dried in a biosafety cabinet and then autoclaved. A more detailed description of this treatment can be found at Schumann-Muck et al. (2023a). In order to produce the coated plucking fingers used in this study, a nanoscale layer of silicon dioxide was applied by Nanopool GmbH (Germany) utilising their commercial product Liquid Glass Metall. Afterwards, the same number of coated and uncoated fingers were inserted into a drum plucker (plucking drum, 92 cm diameter, 42 cm height, equipped with 258 plucking fingers).

Two stainless-steel rods (type 304; X5CrNi1810) with a diameter of 30 mm and a length of 110 cm were custom-manufactured by SKS (Sondermaschinen- und Fördertechnikvertriebs-GmbH, Germany). They were divided into sections of 10 cm in length, and these sections were then alternately left uncoated or coated with a nanoscale layer of silicon dioxide applied by Nanopool GmbH, Germany, using its commercial product Liquid Glass Metall.

One rod was then fixed at a mobile carcass rack for attachment experiments in such a way that it was at a height of 140 cm parallel to the ground. For the detachment experiments, the second rod was attached parallel above the first one at a distance of 6 cm, but offset by one section, so that coated and uncoated sections were directly above each other.

### Study design and recovery of the bacteria

The broilers used for the experiments were purchased already killed from a commercial poultry processor and used for experiments within a maximum of 2 h after killing.

Attachment and detachment tests with plucking fingers were repeated four times each using five uncoated and five coated fingers. Five broilers, taken from slaughter line after stunning and bleeding, were used for every finger experiment. These were scalded for 3 min in tap water at about 53 °C (Brühanlage Typ L1000, Westerhoff Geflügeltechnik GmbH, Germany). After that, each broiler was inoculated using a pipette with 10 mL mixed bacterial suspension, which was distributed over the entire carcass. Then, these five broilers were defeathered together for 30 to 40 s in the drum plucker equipped with coated and uncoated plucking fingers. Afterwards, the carcasses were removed and discarded, and the respective ten plucking fingers to be examined were taken from the machine. The detachment experiments followed subsequently. Therefore, the already used and, thus, contaminated plucking machine was first given a coarse cleaning with tap water at 15 °C for 15 s using a hand shower to soak and loose coarse organic soiling such as feathers. This was followed by the actual cleaning in form of high-pressure cleaning at 130 bar and a water temperature of 75 °C for 1 min (HDS 9/18–4 M, Alfred Kärcher Vertriebs-GmbH, Germany). Recovering bacteria from plucking fingers for attachment and detachment experiments was carried out following the method of Arnold. The tips of the fingers were cut off behind the third rib with sterile scissors, placed in a 50-mL centrifuge tube (centrifuge tube 50, TPP, Switzerland) filled with 10 mL sodium chloride peptone solution and manually shaken for 15 to 20 s. They were kept refrigerated at 5 °C for a maximum of 6 h until further processing. Then decimal dilutions of the liquids were spread on the appropriate selective nutrient media for detection of each of the three bacteria used and incubated for 24 h at the optimal growth temperatures respectively: *E. coli* on Tryptone Bile X Glucuronic Agar (TBX; TN1259; sifin diagnostics GmbH, Germany) at 42 °C, *S*. E. on Xylose Lysine Deoxycholate Agar (XLD; 85,864.5000; VWR International GmbH, Germany) at 37 °C and *C.* *jejuni* on modified Cefaperazone Charcoal Desoxycholate Agar plates (mCCDA, Oxoid GmbH, Germany) microaerobically at 42 °C.

Experiments for attachment to the stainless-steel rod were repeated four times and performed with three broiler carcasses each, which were taken from a slaughter line after plucking. These were inoculated in the breast area using a pipette with 3.0 mL of the bacterial suspension, which was distributed with sterile spatulas. Carcasses were then hooked into a slaughter belt, which moved at a speed of 0.1 m/s. This caused them to move along the stainless-steel rod, thereby touching it at the inoculated breast area. Then, six consecutive sections, and thus alternately coated and uncoated sections, were sampled on the rod. For detachment experiments, a broiler was inoculated in the breast area with 3.0 mL of the bacterial suspension and moved along the rods with the aid of the slaughter belt in such a way that both rods were touched simultaneously with the inoculated breast area. Afterwards, the broiler was inoculated a second time and passed along the rods again. The two rods attached one above the other were then rinsed with 1.5 l water (76–77 °C). For each test, the first touched section of each rod was sampled, and thus a coated and an uncoated section were sampled at the same time. This was repeated 10 times.

According to DIN EN ISO 18593, bacteria were recovered from the rod by sampling each section vertically and horizontally for 30 s by wiping with uniformly strong pressure using a sterile touch sponge (Polywipe with Peptone Saline; Medical Wire and Equipment, UK). The sponge was then placed in a plastic bag filled with 90 mL sodium chloride peptone solution, which was sealed to prevent leakage and kept refrigerated at 5° C for a maximum of 6 h until further processing. Afterwards, the bags were stomached for 1 min at 260 rpm (Stomacher 400 Circulator, Seward Ltd., UK). Decimal dilutions were then prepared from the liquids, which were plated and incubated on the appropriate selective culture media for the bacteria used, as described for the plucking fingers.

### Statistics

Bacterial counts were log_10_ transformed to perform the statistical analysis. Attachment and detachment of *S*. E., *E. coli* and *C. jejuni* from plucking fingers was calculated as the average of four replicates each from the recovered bacterial counts of five fingers per replicate. The attachment and detachment values represent the number of bacteria that were able to attach to the fingers or could not detach from them by the rinsing process. Differences between coated and uncoated plucking fingers per treatment were statistically analysed with an unpaired *t*-test at an alpha level of significance of 0.05.

For attachment of the target organisms to the stainless-steel rod, the section average bacterial counts of four replicates were used to calculate linear regression of fit. Linear regression lines of coated and uncoated steel were compared based on slope and intersect following the procedure described by Glantz (2012) to generate *F* values for reference at an alpha level of significance of 0.05.

Detachment of the bacteria from the stainless-steel rod was calculated as the average of 10 replicates from the recovered bacteria counts of one section per replicate. The detachment values represent the number of bacteria that were able to remain attached to the rod after rinsing. Differences between coated and uncoated rod sections per treatment were statistically analysed using an unpaired *t*-test at an alpha level of significance of 0.05.

Except comparison of linear regression lines, all statistical analyses were carried out using Prism 9 (GraphPad Software, LLC, USA).

## Results

### Plucking fingers

**Fig. 1:**
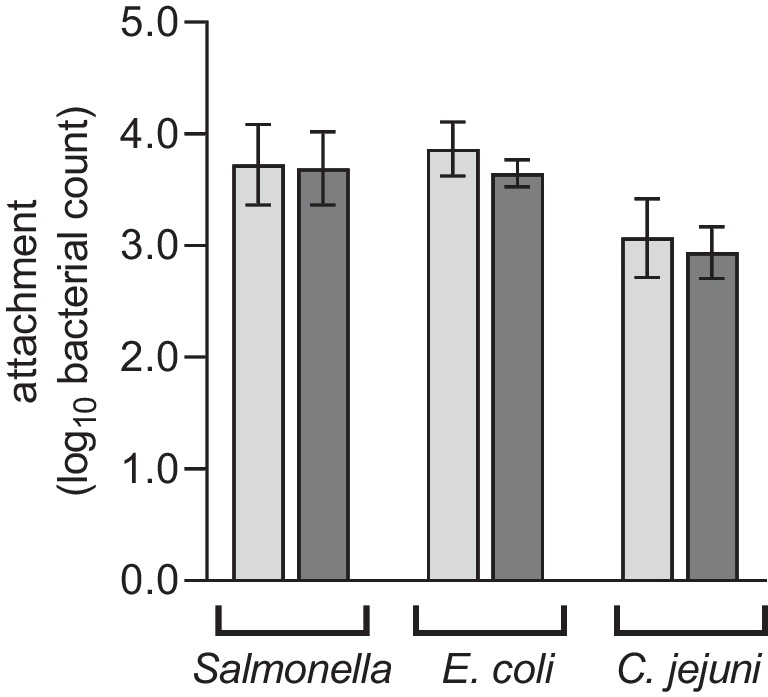
Attachment of *S*. E*., E. coli* and *C.* *jejuni* on uncoated (light grey) and coated (dark grey) plucking fingers. The mean values ± standard deviation from four replicates are shown

Attachment tests on the plucking fingers resulted in *S*. E. attachment of 3.724 log_10_ cfu on uncoated and 3.691 log_10_ cfu on coated fingers. This difference was not statistically significant (*p* = 0.896; Fig. 1). Also, no statistically significant difference (*p* = 0.165) was detected between uncoated and coated plucking fingers for *E. coli* (3.863 log_10_ cfu vs. 3.649 log_10_ cfu; Fig. 1). The same results were observed for *C.* *jejuni* (3.067 log_10_ cfu vs. 2.938 log_10_ cfu; *p* = 0.564; Fig. 1).

**Fig. 2:**
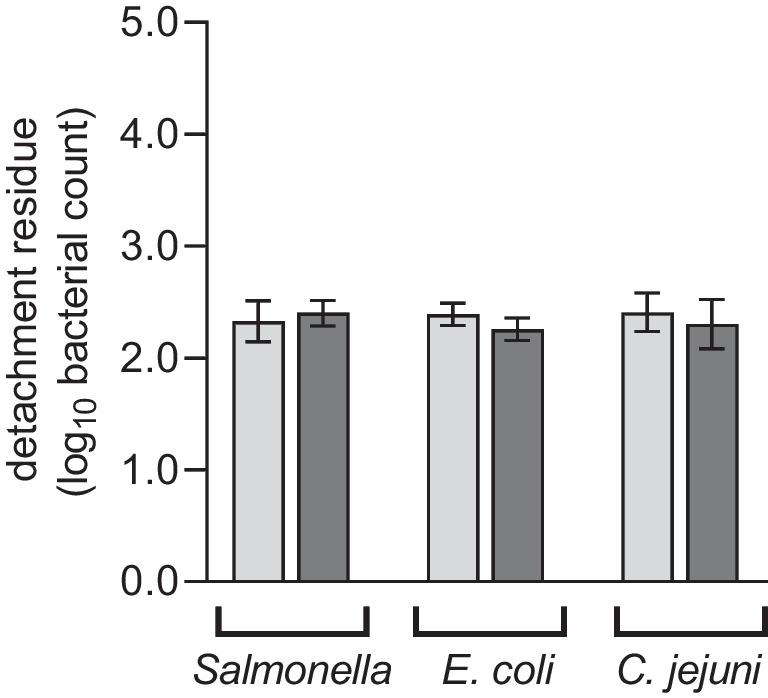
Still attached *S*. E*.*, *E. coli* and *C.* *jejuni on* uncoated (light grey) and coated (dark grey) plucking fingers after detachment procedures with hot water. The mean values ± standard deviation from four replicates are shown

After rinsing, similar numbers of *S*. E. were still adhering to the uncoated and coated plucking fingers (2.327 log_10_ cfu vs. 2.401 log_10_ cfu, respectively, *p* = 0.517; Fig. 2). The same effect was observed for *E. coli* (2.391 log_10_ cfu vs. 2.256 log_10_ cfu, *p* = 0.105; Fig. 2) and *C.* *jejuni* (2.408 log_10_ cfu vs. 2.302 log_10_ cfu, *p* = 0.479; Fig. 2).

### Stainless steel

The mean values for attachment of the respective bacteria on the sections examined on the rod can be taken from Table 1. Due to a smearing effect caused by the carcasses sliding along the rod, linear regression lines were determined from the mean values for the uncoated and coated sections, which were used for the statistical evaluation. Assuming differences in attachment regarding the coating, the regression lines based on bacterial counts on coated or uncoated stainless-steel sections would differ significantly in slope and/or intercept. The procedure mentioned in the data analysis section compares regression lines in regard to these two characteristics, yielding in an *F* value. At an alpha level of significance of 0.05, for *ν*_n_ = 2 and *ν*_d_ = 2, the resulting *F* value needs to exceed 19.00. The regression line for *S*. E for the coated sections was *y* =  − 0.2671*x* + 7.2239 and for the uncoated sections *y* =  − 0.3371*x* + 7.3641. This resulted in an *F* value of 0.987. For *E. coli*, the equation *y* =  − 0.2850*x* + 7.0100 for the uncoated sections and *y* =  − 0.2152*x* + 6.8285 for the coated ones resulted in an *F* value of 1.290 and for *C. jejuni*, the regression line for the coated sections was *y* =  − 0.4557*x* + 6.7429 and for the uncoated sections *y* =  − 0.4562*x* + 6.7243 (*F* value = 0.003). In no case, the *F* value exceeded 19.00, so there were no statistical differences in attachment for any bacteria in regard of coating.

**Table 1:** Attachment ^a^ of *S.* E., *E. coli* and *C.* *jejuni* on uncoated (1;3;5) and silicon dioxide-coated (2;4;6) stainless-steel rod sections

	Sections	*S*. E	*E. coli*	*C. jejuni*
Uncoated	1	6.96	6.68	6.22
	3	6.48	6.25	5.46
	5	5.61	5.54	4.39
Coated	2	6.73	6.43	6.00
	4	6.07	5.90	4.59
	6	5.66	5.57	4.17

^a^Mean values of log bacterial counts from four replicates are shown

**Fig. 3:**
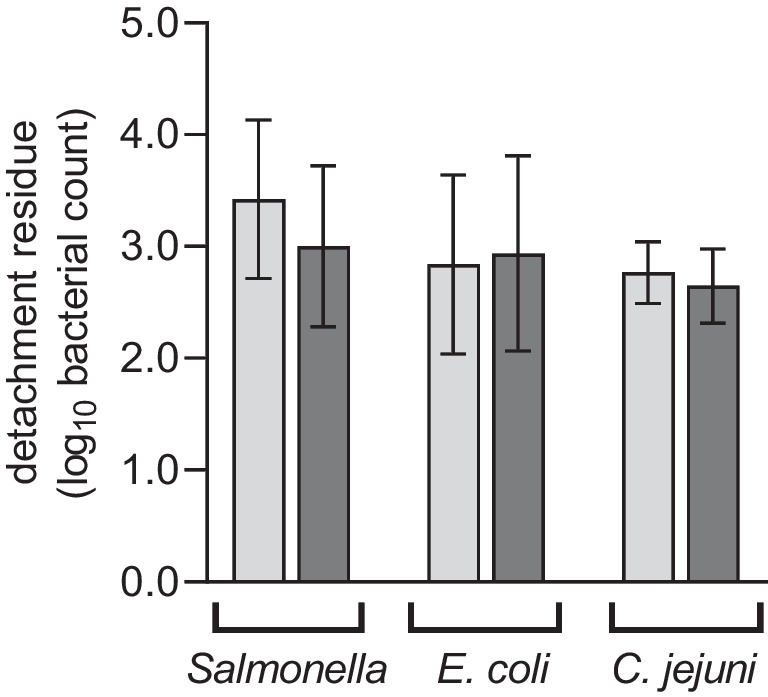
Still attached *S*. E*., E. coli* and *C.* *jejuni on* uncoated (light grey) and coated (dark grey) stainless-steel rod sections after cleaning with hot water. The mean values ± standard deviation from 10 replicates are shown

Regarding detachment, there was a slight difference in *S*. E. between the non-detached bacteria of the uncoated (3.423 log_10_ cfu) and coated (3.002 log_10_ cfu) sections, but this was not found to be statistically significant (*p* = 0.205; Fig. 3). In the case of *E. coli*, no statistically significant difference was found between coated and uncoated sections in the still attached bacteria either (2.840 log_10_ cfu vs. 2.937 log_10_ cfu; *p* = 0.82; Fig. 3). For *C.* *jejuni*, 2.768 log_10_ cfu on the uncoated and 2.646 log_10_ cfu on the coated stainless-steel sections were unable to detach. However, this did not represent a statistically significant difference (*p* = 0.387; Fig. 3).

## Discussion

### Plucking fingers

In this study, silicon dioxide coating of plucking fingers did not result in less attachment or better detachment for *S*. E., *E. coli* and *C.* *jejuni*. Similar results were already reported by Schumann-Muck et al. (2023a) and Blaeske et al. (2023) after examining the same coating on plucking fingers on a laboratory scale. All of these findings are plausible considering the results of Arnold and Silvers (2000), who reported that plucking fingers, due to the rubber used for their manufacture, showed lower bacterial adhesion and, thus, less biofilm formation compared to other materials, e.g. stainless steel. The applied silica coating of the plucking fingers, therefore, does not improve the material properties with regard to the attachment of the bacteria due to the covering of the already bacteria-reducing material.

Despite the already existing laboratory results, the tests were carried out on a pilot plant scale, as a possible influence of the test conditions in the technical centre, such as the use of a plucking machine, could not be fundamentally ruled out. This is particularly important with regard to the use of a plucking machine. One reason why the plucking step is mentioned in several sources as one of the main causes of cross-contamination, despite the bacteria-reducing properties of the plucking finger rubber, may be airborne spread of bacteria via aerosols or water droplets. One source for this could be the water flushing during plucking process, which leads to aerosol formation and, thus, to a greater spread of bacterial contamination even outside of the plucking machine, as Allen et al. (2003) confirmed in their study. They also observed that the bacterial distribution pattern was affected by the motion of the plucking fingers and the design of the plucking machine, because more *E. coli* were found on carcasses after plucking with a contra-rotating machine (mean log_10_ 5.23 ± 0.09 cfu/mL carcass rinse for single pass) than with a disc machine (mean log_10_ 2.43 ± 0.41 cfu/mL carcass rinse), which made the former less effective in removing the marker organism. These considerations must also be taken into account in our study, as a drum plucker operating on the principle of a disc machine was used for our experiments. Due to its construction, it is possible that the contact of the five simultaneously plucked broilers with each other and with the plucking fingers during the 40 s lasting plucking process led to increased contamination of the fingers. The in-process water in combination with the rapid rotation and the closed lid of the machine also led to a strong distribution of water within the plucker and to aerosol formation, which may also have influenced the distribution of bacteria. These points must be taken into account when assessing the test results, as the use of the coated fingers in a plucking belt, which is most commonly used in commercial slaughterhouses, would possibly produce different results compared to the use in the drum plucker.

### Stainless steel

In the present study, nanoscale coating did not significantly reduce bacterial adhesion to stainless-steel rod section. This result is in contrast with the study of Puckett et al. (2010) who found that nanorough titanium surfaces had a statistically significant reduction in attachment of all bacteria they examined compared to conventional titanium (*p* < 0.01). Thus, it is reasonable to reduce bacterial attachment to stressed surfaces by means of modifications. This is also particularly important for stainless steel as was examined in the present study. Although stainless steel is frequently used in the food industry due to its relatively low initial costs and high corrosion resistance (Schmidt et al. 2012), Flint et al. (2000) were able to show that, compared to other surfaces such as glass, stainless steel provides a good substrate for bacteria such as *Streptococcus thermophilus* to adhere to. For this reason, attempts have already been made with different types of stainless-steel surface modification. A nanoscale amorphous SiO_x_C_y_H_z_ coating proved to be successful, as Di Cerbo et al. (2021) were able to demonstrate a 5-log decrease of *E. coli* and *S*. Typhimurium compared to the uncoated surfaces.

One reason for the difference between the results of those studies and the current one could be the contact time of the bacteria with stainless steel. Since the conditions in the present study intended to mimic those in the slaughterhouse, the contact time of inoculated carcasses while sliding along the rod was only a few seconds. In other studies, exposure times ranging from 30 min (Zakarienė et al. 2018) to 1 h (Schlisselberg and Yaron 2013) were chosen for initial bacterial attachment to the surfaces, resulting in reduced attachment on the treated surfaces.

When investigating bacterial detachment from the stainless-steel rod using hot water, no improvement was demonstrated by the coating in the present study. These results are in contrast with the report by Ivanova et al. (2012) who were able to show that cicada wings with a naturally ultra-thin nanostructured topography offer good protection against *Pseudomonas aeruginosa*, as they exhibited a water-repellent activity and thus a kind of self-cleaning effect. The development of surface-modifying coatings based on these findings is often related to the modification of relevant surface properties such as surface free energy, roughness and especially surface wettability. One measure of this is the water contact angle, which, as shown by Schumann-Muck et al. (2023b), increases from 56.7 to 115.4° when the identical silicon dioxide coating was applied to stainless-steel discs. This results in a hydrophobic character of the surface with a lower wettability, which should actually lead to an increased detachment of the bacteria from the coated surface. One reason that the results of the present study are different could be due to the organic material transferred to the rod by the carcasses, whose most important components in this context are fat and proteins. High protein concentrations are able to influence the interaction of bacteria and surfaces (Singh et al. 2011). On the one hand, the proteins can act as a passivating layer and thus lead to a reduction in bacterial adhesion to the nanoscale surface. On the other hand, a flattening effect was observed due to the protein layer formed, which could lead to a reduction in the function of the nanoscale coating. This flattening effect could confirm our results, as the proteins of the chicken carcasses could reduce the hydrophobic effect of the applied nanoscale coating. As a result, the detachment of the bacteria is made more difficult. The results generated in the present study are consistent with previously published results investigating the detachment of various bacteria from stainless steel coated with nanoscale silicon dioxide (Blaeske et al. 2023; Hillig et al. 2024; Schumann-Muck et al. 2023b).

In comparison to previous laboratory-scale experiments, surfaces were contaminated by direct carcass contact. Thus, the coating would rather affect the contamination of organic material (including the bacteria) than the bacteria itself when applied as a suspension. The area of organic contamination was not always recognisable on stainless-steel rods and not at all on plucking fingers. Furthermore, a greater area than only the contacting area was sampled. Here, also bacterial suspension could have been included, which was eventually spilled onto the surface due to the carcass motion (especially in the plucking machine). Therefore, the results would represent a combined effect on solely bacterial adhesion/detachment and on organic material contamination and cleaning.

It must be noted that it is questionable whether the results of this study can be generalised for the bacteria used, as only one strain of each bacterium was used. Especially in the case of *Campylobacter*, it is known that there are differences between strains, for example with regard to colonisation ability and survivability (Park 2002). For this reason, the results presented here are difficult to transfer to other strains.

## Conclusion

In the current study, the effects of a nanoscale silica coating on the attachment and detachment of *S*. E., *E. coli* and *C. jejuni* were investigated on a pilot scale by comparing coated and uncoated plucking fingers and stainless-steel surfaces.

The coating did not lead to reduced attachment or better detachment of the bacteria strains examined regardless of the examined surface material.

In order to reduce the bacterial contamination of chicken meat, the modification of slaughterhouse surfaces particularly affected by cross-contamination with the aid of coatings could represent a possible approach. However, as can be seen from the available results, the silicon dioxide coating investigated here cannot be recommended for use at the present time for the purpose of reducing *S*. E., *E. coli* and *C. jejuni* in slaughterhouses. Since in general, a bacteria-reducing effect has been described for several nanoscale coatings, several parameters of such coatings should be focussed in future research to achieve a potential effect for the respective bacteria in the poultry slaughterhouse environment. Such parameters could include the coating material as well as the structure and formation of the nanolayer, or the resulting roughness. Analyses of durability of the coating could also be useful from an application perspective.

## Data Availability

The datasets used and/or analysed during the current study are available from the corresponding author on reasonable request.
